# Dioscin Promotes Prostate Cancer Cell Apoptosis and Inhibits Cell Invasion by Increasing SHP1 Phosphorylation and Suppressing the Subsequent MAPK Signaling Pathway

**DOI:** 10.3389/fphar.2020.01099

**Published:** 2020-07-24

**Authors:** Shuyun He, Jinrui Yang, Shaobo Hong, Haijian Huang, Qingguo Zhu, Liefu Ye, Tao Li, Xing Zhang, Yongbao Wei, Yunliang Gao

**Affiliations:** ^1^ Department of Urology, The Second Xiangya Hospital, Central South University, Changsha, China; ^2^ Department of Urology, The People’s Hospital of Xiangtan Country, Xiangtan, China; ^3^ Shengli Clinical Medical College of Fujian Medical University and Department of Urology, Fujian Provincial Hospital, Fuzhou, China; ^4^ Shengli Clinical Medical College of Fujian Medical University and Department of Pathology, Fujian Provincial Hospital, Fuzhou, China; ^5^ Department of Urology, The Traditional Chinese Medicine Hospital of Yangzhou, Yangzhou University of Traditional Chinese Medicine, Yangzhou, China

**Keywords:** dioscin, prostate cancer, cell apoptosis, cell invasion, SHP1, mitogen-activated protein kinase

## Abstract

Dioscin possesses antioxidant effects and has anticancer ability in many solid tumors including prostate cancer (PCa). Nevertheless, its effect and mechanism of anti-PCa action remain unclear. The tyrosine protein phosphatase SHP1, which contains an oxidation-sensitive domain, has been confirmed as a target for multicancer treatment. Further studies are needed to determine whether dioscin inhibits PCa through SHP1. We performed *in vitro* studies using androgen-sensitive (LNCaP) and androgen-independent (LNCaP -C81) cells to investigate the anticancer effects and possible mechanisms of dioscin after administering interleukin-6 (IL-6) and dihydrotestosterone (DHT). Our results show that dioscin inhibited cell growth and invasion by increasing SHP1 phosphorylation [p-SHP1 (Y536)] and inhibiting the subsequent P38 mitogen-activated protein kinase signaling pathway. Further *in vivo* studies confirmed that dioscin promoted caspase-3 and Bad-related cell apoptosis in these two cell lines. Our research suggests that the anticancer effects of dioscin on PCa may occur through SHP1. Dioscin may be useful to treat androgen-sensitive and independent PCa in the future.

## Introduction 

Interleukin-6 (IL-6) and androgen are two important factors that induce the occurrence and progression of prostate cancer (PCa) through activation of the P38 mitogen-activated protein kinase (MAPK) (extracellular regulated protein kinases 1/2, Erk1/2), phosphoinositide 3-kinase/protein kinase B (PI3K/AKT) and other signaling pathways to induce cancer cells and malignant transformation (Schafer and Brugge, 2007; Mukherjee and Mayer, 2008; Erdogan et al., 2018; Johnson et al., 2018). Various plant extracts, such as dioscin, possess anticancer effects (Kim et al., 2014; Tao et al., 2018). Dioscin has antiinflammatory, immunomodulatory, hypolipidemic, antiviral, antifungal and antiallergic effects, and also shows anticancer antioxidant activity (Tao et al., 2018). Dioscin has significant antitumor effects and inhibits a variety of solid tumors (Wei et al., 2013; Chen et al., 2014; Kim et al., 2014; Tao et al., 2017; Zhou et al., 2019). Two studies have confirmed that dioscin promotes caspase-3 and Bcl-2-associated cell apoptosis and exhibits anticancer effects on PCa. These studies demonstrated that the anti-PCa effect of dioscin occurs *via* activation of estrogen receptor-beta (Chen et al., 2014; Tao et al., 2017). However, the role and mechanism of dioscin in PCa have not yet been fully elucidated. This study aimed to decipher the mechanism underlying the effect of dioscin on PCa.

SHP1, a member of the protein tyrosine phosphatase family, reversibly oxidizes active-site cysteine residues to sense reactive oxygen species and affect tyrosine phosphorylation-mediated cellular processes (Tonks, 2005; Dustin et al., 2019). Previous studies have reported that SHP1 is overexpressed in PCa cells (Wu et al., 2003) and that SHP1 knockdown causes cell-cycle arrest in the PC3 human prostate cancer cell line (Rodriguez-Ubreva et al., 2010). Another study confirmed that SHP1 predicts outcome after radical prostatectomy (Tassidis et al., 2010a). These results indicate that SHP1 may be a promising target to treat PCa and demonstrate that SHP1 is activated by plant extracts (Pesce et al., 2015). As dioscin is an herbal component, whether it activates SHP1 and plays a role in PCa deserves further investigation.

## Materials and Methods

### Cell Culture and Transfection

LNCaP-C-33 (LNCaP) and LNCaP-C81 are androgen-sensitive and androgen-independent PCa cells, respectively (Igawa et al., 2002; Muniyan et al., 2015). The cells used in this study were purchased from the Chinese Academy of Sciences (Beijing, China). Cells were routinely cultured in a conventional medium containing phenol red-positive RPMI 1640 medium supplemented with 10% (v/v) fetal bovine serum (FBS), 2 mm glutamine, and 50 μg/ml gentamicin. Lipofectamine™ 2000 (Invitrogen, Carlsbad, CA, USA) was used to transfect small interfering RNA (siRNA). The diluted siRNA was mixed with Lipofectamine™ 3000 and the resulting siRNA-Lipofectamine™ 3000 complex was added to wells containing cells and culture medium for cell transfection. The four SHP1 siRNA sequences used were as follows:

SiR-1 (siRNA-893, F: 5’-GGUGAAUGCGGCUGACAUUTT-3’, R: 5’-AAUGUCAGCCGCAUUCACCTT-3’);SiR-2 (siRNA-666 F: 5’-CCUGGAGACUUCGUGCUUUTT-3’, R: 5’-AAAGCACGAAGUCUCCAGGTT-3’); SiR-3 (siRNA-340, F: 5’-GCAAGAACCAGGGUGACUUTT-3’, R: 5’-AAGUCACCCUGGUUCUUGCT-3’); SiR-NC (negative control, F: 5’-UUCUCCGAACGUGUCACGUTT-3’, R: 5’-ACGUGACACGUUCGGAGAATT-3’).

### Cell Proliferation

A 100-μl aliquot of cells (about 1 × 10^4^ cells) was added to each well of a 96-well plate and placed in a 37°C 5% CO_2_ incubator for 24 h. An appropriate concentration of the drug was added and incubated. Then, 5× 3-(4,5-dimethylthiazol-2-yl)-2,5-diphenyltetrazolium bromide (MTT; Sigma, St. Louis, MO, USA) was diluted to 1× MTT with Dilution Buffer, and 50 μl was added to each well followed by a 4-h incubation. The supernatant was aspirated and 150 μl of dimethyl sulfoxide (Sigma) was added to each well. A microplate reader (Molecular Devices Sunnyvale, CA, USA) was used to detect the optical density of each well at 570 nm, and the cell survival rate was calculated.

### Cell Apoptosis Assay

Annexin V-fluorescein isothiocyanate/propidium iodide (PI) (Mbchem M3021, Mumbai, India) was used to detect cell apoptosis. The cells were collected at room temperature, resuspended in 50 μl of prechilled 1× PBS (4°C) and centrifuged. Each sample (10^5^–10^6^ cells) was prepared with 100 μl of Annexin-V labeling solution. The cells were suspended and incubated for 15 min, and then 10 μl of PI was added. A prepared dilution (cold 400 μl of Binding Buffer) was added to 100 μl of incubation solution, and flow cytometry was performed within 15 min.

### Cell Scratch Wound Repair Assay

A reference line was drawn on the back of a six-well plate, and 5 × 10^5^ cells were added to each well. After the cells covered the bottom of the well, a 20-µl pipette tip was used to create an “I”-shaped scratch in the middle of the well as the 0-h control. Each well was washed three times with serum-free medium to remove the scratched cells. The plate was placed in a 5% CO_2_ incubator at 37°C for 24 h and then removed to record relative scratch width.

### Cell Formation Assay

Monolayer cells cultured in the logarithmic growth phase were digested with 0.25% trypsin, pipetted into single cells and counted. The cell concentration was adjusted to 1,000 per dish, and the drug was added. The cell suspension was inoculated into a dish containing 3 ml of 37°C prewarmed culture medium and gently rotated to uniformly disperse the cells. The cells were incubated at 37°C at 5% CO_2_ with saturated humidity for 2–3 weeks. The number of cells contained in each dish was counted, and the cell formation rate was calculated.

### Cell Invasion Assay

The lower and upper wells of a Transwell plate (Corning, Corning, NY, USA) were coated with a 60–80 μl dilution of Matrigel. The Matrigel was polymerized into a gel at 37°C for 30 min to prepare the Transwell plate. After the cells were digested, centrifuged, washed with PBS and resuspended, cell density was adjusted to 5 × 10^4^/ml. Next, 1 ml of FBS-containing medium was added to the lower well and the cell suspension was added to the upper well. The cells were cultured for 24 h and the basement membrane of the lower well was removed. The cells were wiped off the Matrigel and the upper well was fixed with 95% alcohol for 15–20 min and stained with hematoxylin for 10 min. The cells were then counted under an inverted microscope.

### Tumor Formation Assay in Nude Mice

Normal cultured LNCaP and LNCaP-C81 cells were subgrouped and processed as follows: LNCaP-C81 tumor formation without any special treatment (group A: L-C81); LNCaP-C81 cells treated with the optimal dioscin dose (Cat. No.: HY-N0124, Purity: 98.33%, MedChemExpress LLC, Monmouth Junction, NJ, USA) (group B: L-C81 + D); SHP1 knockdown LNCaP-C81 cells treated with the same dioscin dose (Group C: L-C81 + KD + D); LNCaP tumor formation without any special treatment (Group D: LNCaP); LNCaP treated with the optimal dioscin dose (Group E: LNCaP + D); and SHP1 knockdown LNCaP cells treated with the same dioscin dose (Group F: LNCaP + KD + D). Each group of cells was adjusted to 1 × 10^6^ cells. A cell suspension with a total volume of 0.2 ml was inoculated at each injection point. Tumor formation was induced near the extremities of 6-week-old BALB/c nude mice (purchased from Chinese Academy of Sciences). After 1 week of rearing, tumor size was measured (longest diameter: a; and shortest diameter: b; once every 3 days, six times in a row). A tumor growth curve was plotted, with time as the abscissa and tumor volume as the ordinate (tumor volume was calculated using the formula: v = a × b^2^ × 0.52; volume unit: mm^3^). Tumor tissues were obtained to detect Ki67 by immunohistochemistry and other proteins were also analyzed.

### Western Blot

A radioimmunoprecipitation assay (RIPA) lysate [containing 1% NP-40, 0.1% SDS (Sigma), and 50 mM DTT] was used to extract the cell and tissue proteins. The proteins were separated by 10% sodium dodecyl sulfate-polyacrylamide gel electrophoresis, and 1 ml of primary antibody (**Supplementary Table 1**) was added. The primary antibody was discarded after a 2-h incubation. The gel was washed with 2–3 ml PBST followed by 1 ml of secondary antibody (**Supplementary Table 1**), and the gel was incubated at 4°C overnight. The gel signals were analyzed with Gel pro version 4.0 software (Media Cybernetics, Silver Spring, MD, USA).

### Immunohistochemical Staining

Tissue sections were sliced at a thickness of 3 µm and baked at 60–65°C for 4 h. After the sections were dewaxed and hydrated, they were washed with 1× PBS buffer (0.01 M, pH 7.2). The paraffin section tissue antigen was repaired using the high-pressure method and the sections were rinsed with PBS. Next, 50 μl of primary antibody (Ki-67 GTX16667/rabbit antibody, dilution 1: 100; GeneTex Inc., Irvine, CA, USA) was added to each section, followed by an overnight incubation at 4°C. After washing with PBS, 50 μl of biotin-labeled secondary antibody was sequentially added and 50 μl of streptavidin-peroxidase solution was added for the incubation. After washing with PBS, 1–2 drops of freshly prepared 3,3′-diaminobenzidine (Cat. No: DAB-0031/1031) was added to each section and the tissues were observed under a microscope. After counterstaining with hematoxylin, dehydration through an alcohol gradient and neutral gum mounting, each section was analyzed at 200× and 400× magnification.

### Statistical Analysis

Each set of experiments was performed in duplicate or triplicate in accordance with the experimental design and repeated at least two or three times. Representative data from one of three independent experiments were selected and shown in the figures. The average and standard error values of the experimental results were calculated. Student’s *t*-test was used to compare the groups. A p-value < 0.05 was considered significant.

## Results

### Screening for the Optimal Dioscin Dose and Duration

LNCaP-C81 cells were treated with different dioscin concentrations (0.1, 0.5, 1, 5, 10, 25, and 50 μg/ml) for 24 h. The cells were collected to detect SHP1 and its three known phosphorylation sites, namely two C-terminal tyrosine residues (Y536 and Y564) and serine 591 (S591) (Choi et al., 2017; Mkaddem et al., 2017). The results showed that the expression levels of SHP1 and p-SHP1 (Y536) were highest in LNCaP-C81 cells after treatment with 10 μg/ml dioscin for 24 h (**Figures S1A, B**) (p < 0.001). This finding indicates that p-SHP1 (Y536) could be used to measure SHP1 phosphorylation. Then, 10 μg/ml dioscin was applied for different durations (0, 3, 6, 12, 24, and 48 h). The expression of the SHP1 protein and p-SHP1 (Y536) was highest after 24 h of treatment (**Figures S1C, D**) (p < 0.001). Cell function experiments were performed, and the results showed that proliferation of LNCaP-C81 cells was significantly inhibited (**Figure S1E**) and cell apoptosis increased (**Figures S1F, G**) (both p < 0.05) after treatment with 10 μg/ml dioscin for 24 h. Thus, in subsequent experiments, LNCaP-C81 cells were treated with 10 μg/ml dioscin for 24 h.

The same methods were used with LNCaP cells to determine the optimal concentration and duration of dioscin treatment. The expression of the SHP1 protein and p-SHP1 (Y536) was highest after treatment with 10 μg/ml dioscin for 24 h (p < 0.001) (**Figures S2A–D**). Subsequently, the proliferation of LNCaP1 cells was significantly lower (**Figure 2E**) and the rate of cell apoptosis was relatively higher (**Figures S2F, G**) (both p < 0.05). Thus, we selected the 24-h treatment with 10 μg/ml dioscin for subsequent LNCaP cell studies.

### Dioscin Regulates SHP1 to Reverse IL-6-Induced Proliferation and Invasion of PCa Cells

We determined the optimal SHP1 interfering sequence. We transfected LNCaP-c81 with the SiR-NC, SiR-1, SiR-2, and SiR-3 sequences and measured SHP1 expression 24 h later by western blotting. As a result, SiR-2 completely blocked the expression of SHP1, reducing the expression level to 0. Therefore, SiR-2 was considered the optimal SHP1 interfering sequence (**Figures S3A, B**).

**Figure 1:**
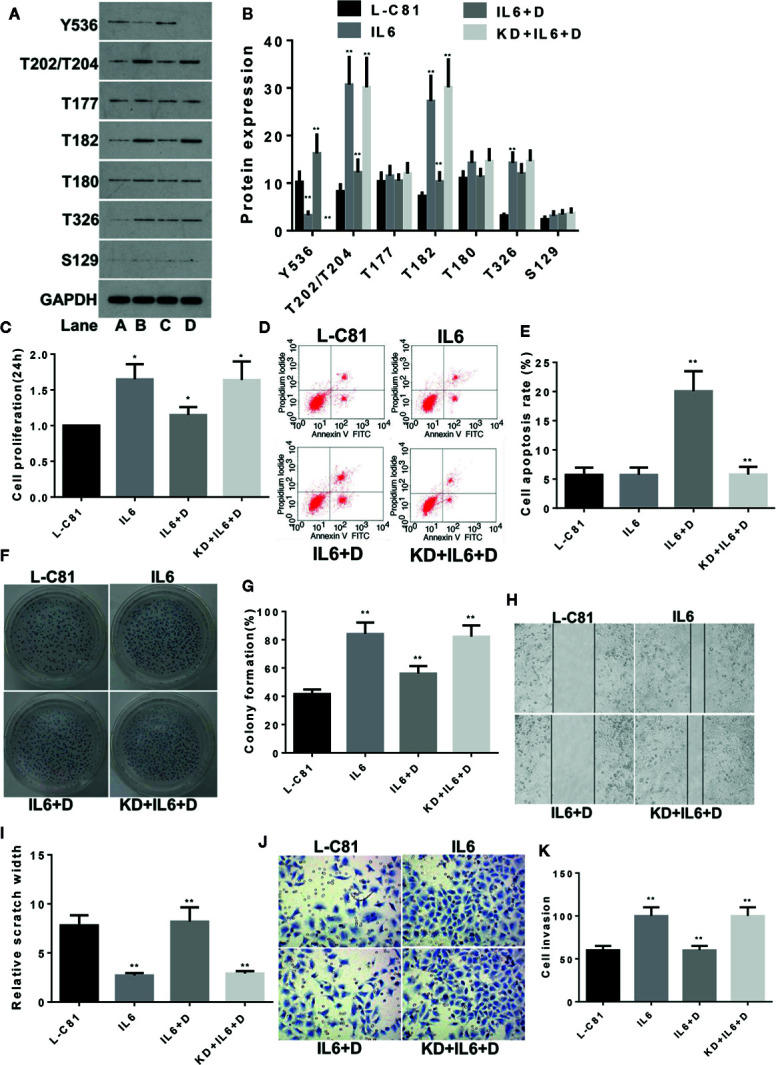
Dioscin regulates SHP1 to reverse IL-6-induced LNCaP-c81 cell proliferation and invasion. **(A, B)** IL-6 significantly inhibits p-SHP1 (Y536) expression and increases the subsequent expression of the p-Erk1/2 (T202/T204), p-P38 (T182) and p-AKT (T326) proteins (all p < 0.01). Dioscin reversed the regulatory effect of IL-6 on these phosphorylated proteins. After inhibiting p-SHP1 (Y536), IL-6 promoted LNCaP-C81 cell proliferation **(C)** and formation **(F, G)** and increased cell wound repair **(H, I)** and migration **(J, K)** (all p < 0.01). However, it had no effect on cell apoptosis **(D, E)**. Dioscin reversed all of the effects of IL-6 on protein regulation and cell functions (all p < 0.05). Representative data from one of three independent experiments are shown in Figure 1 **(A, D, F, H, J)**. * on behalf of p < 0.05; ** on behalf of p < 0.01.

LNCaP-C81 was divided into four groups: routine culture (group A: L-C81); IL-6 intervention (group B: IL6); IL-6 and dioscin co-treatment (group C: IL6 + D) and SHP1 knockdown and co-treatment with IL-6 and dioscin (group D: KD + IL6 + D). The IL-6 dosage for these groups was set to 5 ng/ml (Hobisch et al., 2001). After a 24 h treatment, IL-6 significantly inhibited p-SHP1 (Y536) expression (p < 0.01) and the protein phosphorylation levels of subsequent signaling pathways were also significantly upregulated. The expression levels of p-Erk1/2 (T202/T204), p-P38 (T182) and p-AKT (T326) increased significantly (all p < 0.01). However, the inhibitory effects on p-Erk1/2(T177) and p-P38 (T180) were not significant (all p > 0.05) (**Figures 1A, B**). Dioscin reversed the regulatory effects of IL-6 on these phosphorylated proteins. Dioscin also significantly promoted the expression of p-SHP1 (Y536) (p < 0.01) and inhibited the proteins in the signaling pathways, specifically p-Erk1/2 (T202/T204), p-P38 (T182) and p-AKT (T326) (all p < 0.05) (**Figures 1A, B**). Cell function experiments revealed that IL-6 promoted LNCaP-C81 cell proliferation (**Figure 1C**) and formation (**Figures 1F, G**) after inhibiting p-SHP1 (Y536), and increased the capability of the cells for wound repair (**Figures 1H, I**) and migration (**Figures 1J, K**) (all p < 0.01). However, IL-6 had no significant effect on apoptosis (**Figures 1D, E**) (p > 0.05). In contrast, dioscin promoted the expression of p-SHP1 (Y536), inhibited cell proliferation (**Figure 1C**), increased apoptosis (**Figures 1D, E**), inhibited cell formation (**Figures 1F, G**) and reduced cell wound repair (**Figures 1H, I**) and cell migration ability (**Figures 1J, K**) (all p < 0.05). However, when SHP1 was knocked down, the effect of dioscin was significantly suppressed, suggesting that SHP1 may be a target gene of dioscin.

**Figure 2:**
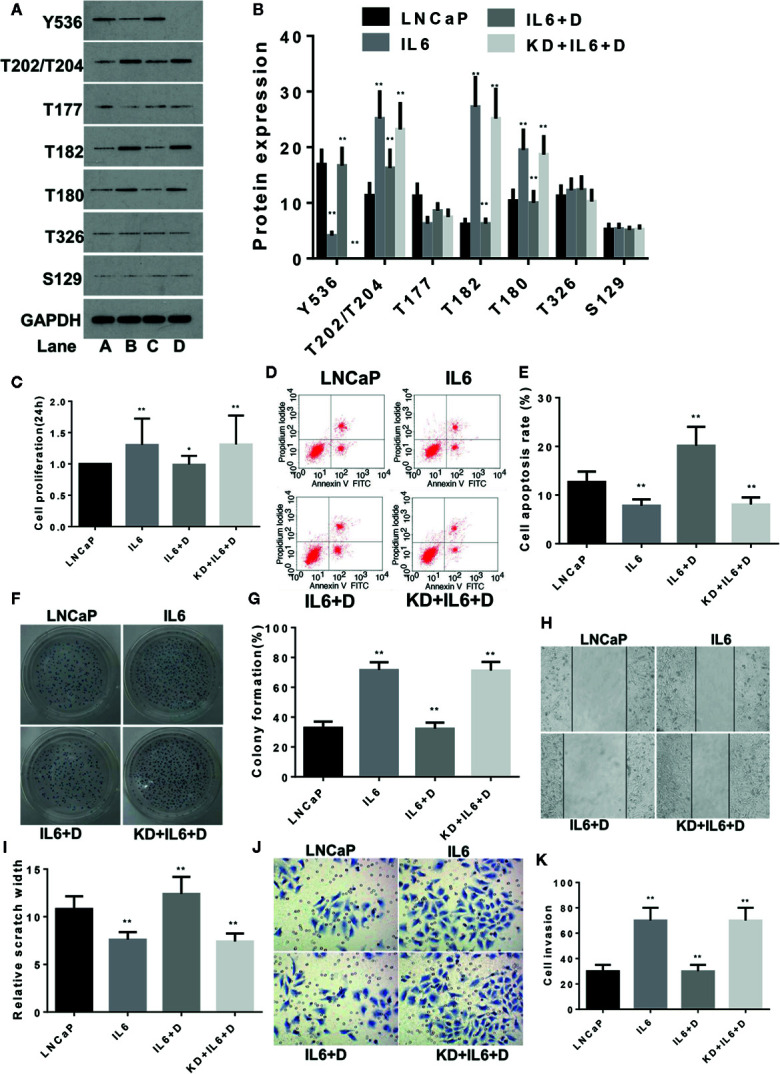
Dioscin regulates SHP1 to reverse IL-6-induced LNCaP cell proliferation and invasion. **(A, B)** IL-6 significantly inhibits p-SHP1 (Y536) expression and increases the subsequent expression of the p-Erk1/2 (T202/T204), p-P38 (T182) and p-P38 (Tyr180) proteins (all p < 0.01). Dioscin reversed the regulatory effect of IL-6 on these phosphorylated proteins. After inhibiting p-SHP1 (Y536), IL-6 promoted LNCaP cell proliferation **(C)** and formation **(F, G)**, reduced apoptosis **(D, E)** and increased wound repair **(H, I)** and migration **(J, K)** (all p < 0.01). Dioscin reversed all of the IL-6 effects on protein regulation and cell functions (all p < 0.05). Representative data from one of three independent experiments are shown in Figure 2 **(A, D, F, H, J)**. * on behalf of p < 0.05; ** on behalf of p < 0.01.

The same method was used to study the effects of IL-6 and dioscin on LNCaP cells. SiR-2 completely blocked SHP1 expression in LNCaP cells (**Figures S4A, B**) (p < 0.01). Further study revealed similar results as shown in LNCaP-C81 cells. IL-6 inhibited p-SHP1 (Y536) expression (p < 0.01), promoted expression of the signaling proteins p-Erk1/2 (T202/T204), p-P38 (T182) and p-P38 (Tyr180) (**Figures 2A, B**) (all p <0.01), promoted cell proliferation (**Figure 2C**), inhibited apoptosis (**Figures 2D, E**), promoted cell formation (**Figures 2F, G**) and enhanced wound repair capability (**Figures 2H, I**) and migration (**Figures 2J, K**) (all p < 0.01). Dioscin reversed these effects of IL-6 in LNCaP1 cells and the effects of dioscin were significantly suppressed after SHP1 knockdown (all p < 0.05).

### Dioscin Regulates SHP1 to Reverse Dihydrotestosterone (DHT)-Induced Proliferation and Invasion by PCa Cells

**Figure 3:**
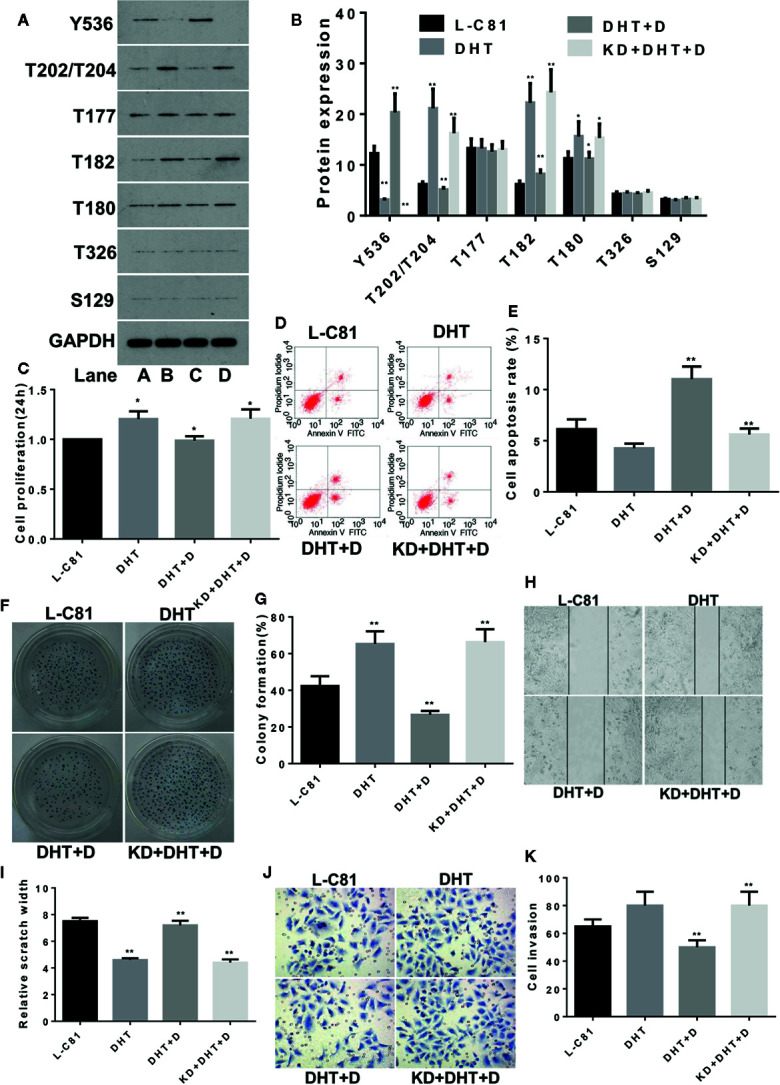
Dioscin regulates SHP1 to reverse DHT-induced LNCaP-C81 cell proliferation and invasion. **(A, B)** DHT significantly reduces p-SHP1 (Y536) expression and increases subsequent expression of the p-Erk1/2 (T202/T204), p-P38 (T182) and p-P38 (T180) proteins (all p < 0.01). Dioscin reversed these effects of DHT on protein regulation in LNCaP-C81 cells (all p < 0.05). After inhibiting p-SHP1 (Y536) expression, DHT significantly promoted LNCaP-C81 cell proliferation **(C)**, formation **(F, G)** and wound repair ability **(H, I)** (all p < 0.05). However, it had no significant effect on cell apoptosis **(D, E)** or migration **(J, K)** (all p > 0.05). Dioscin promoted p-SHP1 (Y536) expression and reversed DHT-induced LNCaP-C81 cell functions (all p < 0.05). Representative data from one of three independent experiments are shown in Figure 3 **(A, D, F, H, J)**. * on behalf of p < 0.05; ** on behalf of p < 0.01.

LNCaP-C81 cells were subjected to the following treatments: conventional culture (group A: L-C81), DHT intervention (group B: DHT), co-treatment with DHT and dioscin (group C: DHT + D) and SHP1 knockdown with DHT and dioscin co-treatment (group D: KD + DHT + D). The dosage and duration of DHT were in accordance with a previous report (Krongrad et al., 1991). After 24 h of treatment, p-SHP1 (Y536) expression decreased significantly after DHT was added (p < 0.01). The proteins for the subsequent signaling pathways, such as p-Erk1/2 (T202/T204), p-P38 (T182) and p-P38(T180), increased significantly (all p < 0.01), but p-Erk1/2 (T177) and p-AKT (T326) levels were not significantly inhibited (all p > 0.05) (**Figures 3A, B**). Dioscin reversed the effects of DHT on LNCaP-C81 cells. Dioscin significantly promoted the expression of p-SHP1 (Y536) (p < 0.01) and inhibited the expression of p-Erk1/2 (T202/T204), p-P38 (T182) and p-P38 (T180) (all p < 0.05) (**Figures 3A, B**). Cell function experiments revealed that after inhibiting p-SHP1 (Y536) expression, DHT significantly promoted LNCaP-C81 cell proliferation (**Figure 3C**), formation (**Figures 3F, G**) and wound repair ability (**Figures 3H, I**) (all p < 0.05). However, it had no significant effect on cell apoptosis (**Figures 3D, E**) or migration (**Figures 3J, K**) (both p > 0.05). Dioscin substantially promoted p-SHP1 (Y536) expression, inhibited cell proliferation (**Figure 3C**), promoted apoptosis (**Figures 3D, E**), inhibited cell formation (**Figures 3F, G**) and reduced wound repair ability (**Figures 3H, I**) and cell migration (**Figures 3J, K**) (all p < 0.05). Similarly, the effect of dioscin was significantly suppressed when SHP1 was knocked down.

**Figure 4:**
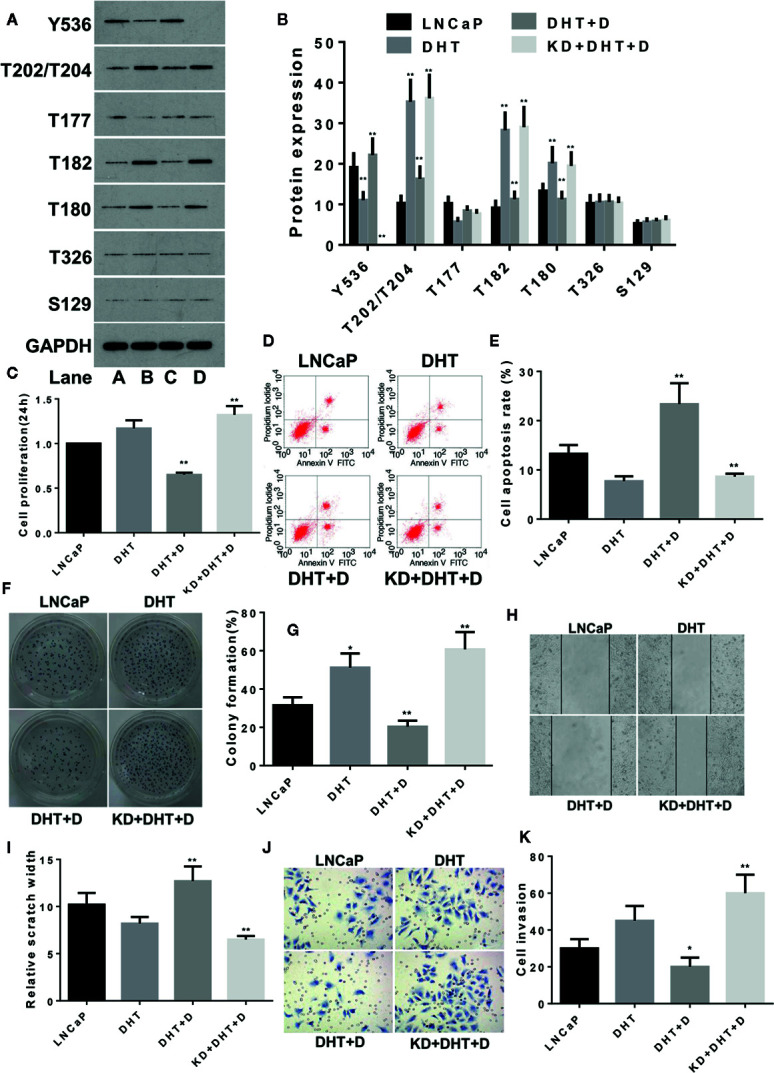
Dioscin regulates SHP1 to reverse DHT-induced LNCaP cell proliferation and invasion. **(A, B)** DHT significantly reduces p-SHP1 (Y536) expression and increases subsequent expression of the p-Erk1/2 (T202/T204), p-P38 (T182) and p-P38 (T180) proteins (all p < 0.01). Dioscin reversed these effects of DHT on protein regulation in LNCaP cells (all p < 0.01). After inhibiting p-SHP1 (Y536) expression, DHT significantly promoted LNCaP cell formation **(F, G)** (p < 0.05), but had no significant effect on cell proliferation **(C)**, apoptosis **(D, E)**, wound repair ability **(H, I)** or migration **(J, K)** (p > 0.05). Dioscin promoted p-SHP1 (Y536) expression and reversed the DHT-induced LNCaP cell functions (all p < 0.05). Representative data from one of three independent experiments are shown in Figure 4 **(A, D, F, H, J)**. * on behalf of p < 0.05; ** on behalf of p < 0.01.

The same method was used to study the effects of DHT on LNCaP cells and similar results were obtained as with LNCaP-C81. DHT inhibited the expression of p-SHP1 (Y536) (p < 0.01) and promoted the expression of p-Erk1/2 (T202/T204), p-P38 (T182), and p-P38 (Tyr180) (all p < 0.01) (**Figures 4A, B**), which in turn promoted cell formation (**Figures 4F, G**) (p < 0.05). DHT had no significant effect on cell proliferation (**Figure 4C**), apoptosis (**Figures 4D, E**), wound repair (**Figures 4H, I**) or migration (**Figures 4J, K**) (all p > 0.05). Dioscin reversed the effects of DHT on these phosphorylated proteins and significantly inhibited cell proliferation, promoted apoptosis, inhibited cell formation and reduced wound repair ability and cell migration (all p < 0.05). The effect of dioscin was consistently reduced significantly when SHP1 was knocked down.

### In Vivo Experiments to Verify the Inhibitory Effects of Dioscin on PCa

**Figure 5:**
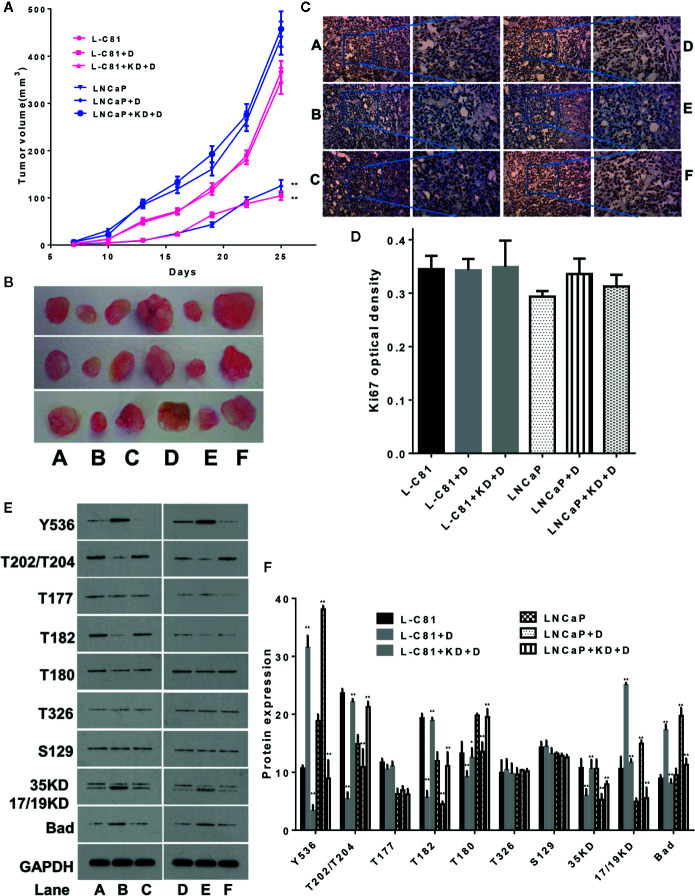
*In vivo* experiments to verify the inhibitory effect of dioscin on prostate cancer. **(A, B)** Dioscin inhibits tumor growth in BALB/c nude mice after tumor implantation with LNCaP-C81 or LNCaP cells (both p < 0.01). **(C, D)** Ki67 was used to assess tumor cell proliferation but did not change significantly between the groups (p > 0.05). **(E, F)** Dioscin promoted the expression of p-SHP1 (Y536) (p < 0.05) and inhibited p-Erk1/2 (T202/T204), p-P38 (T182), p-P38 (T180) and caspase-3 (35 KD) protein expression (all p < 0.01). It also significantly promoted the expression of caspase-3 (17/19KD) and Bad (all p < 0.01). Representative data from one of three independent experiments are shown in Figure 5 **(C, E)**. * on behalf of p < 0.05; ** on behalf of p < 0.01.

After grouping BALB/c nude mice according to the methods described above, dioscin inhibited the growth of nude mice tumors consisting of LNCaP-C81 and LNCaP cells (**Figures 5A, B**) (both p < 0.01). Ki67 expression in tumor tissues was detected by immunohistochemical staining to assess tumor cell proliferation (Gerdes et al., 1984). The expression of Ki67 among the above groups did not change significantly (**Figures 5C, D**) (p > 0.05), suggesting that dioscin may not inhibit tumor cell proliferation *in vivo*. Phosphorylated protein detection revealed that dioscin promoted the expression of p-SHP1 (Y536) (p < 0.05) and inhibited the expression of p-Erk1/2 (T202/T204), p-P38 (T182), p-P38(T180), and caspase-3(35KD) proteins (**Figures 5E, F**) (all p < 0.01), but significantly promoted the expression of caspase-3 (17/19KD) and Bad (**Figures 5E, F**) (both p < 0.01). Dioscin had no significant effects on the expression of p-Erk1/2 (T177), p-AKT (T326), or p-AKT (S129) (all p > 0.05). The effect of dioscin was significantly reduced after SHP1 was knocked down. Correspondingly, the expression of p-SHP1 (Y536) and its subsequent pathway proteins showed the opposite results. These results suggest that dioscin has potential as a targeted drug to promote SHP1 phosphorylation and promote caspase-3 and Bad-related apoptosis by inhibiting the downstream Erk1/2 and P38 signaling pathways (Gajewski and Thompson, 1996; Porter and Janicke, 1999); thus, presenting antitumor effects on PCa.

## Discussion

Erk1/2 is an important subfamily of MAPKs, which control a wide range of cellular activities and physiological processes (Lu and Xu, 2006). MAPKs control the activity or abundance of BCL-2 protein family members (such as Bad) to promote cell survival (Balmanno and Cook, 2009). In addition, the MAPK signaling pathway is closely related to cancer progression (Wagner and Nebreda, 2009; Cuadrado and Nebreda, 2010) and activation of caspase-3 or Bad promotes cell apoptosis (Gajewski and Thompson, 1996; Porter and Janicke, 1999; Min et al., 2004). Dioscin increased apoptosis in PCa cells by promoting the expression of phosphorylated SHP1-p-SHP1 (Y536), inhibiting the expression of p-Erk1/2 (T202/T204), p-P38 (T182) and p-P38(T180) and promoting the expression of apoptosis-related proteins caspase-3 (17/19KD) and Bad. Our study may provide a basis for understanding the mechanism of PCa development and exploring new therapeutic drugs for the disease.

IL-6 plays an important role in both the progression and treatment of PCa (Hobisch et al., 2001; Johnson et al., 2018). IL-6 is a pleiotropic pro-inflammatory cytokine involved in the development of PCa and plays a role mediated through autocrine and paracrine mechanisms (Adekoya and Richardson, 2020). Long-term application of IL-6 promotes PCa cell proliferation and thus can be used to establish *in vitro* models of advanced PCa (Hobisch et al., 2001). Several reports indicate that IL6 is produced by invasive PCa and the PC3 and DU145 cell lines, while its production is low or non-existent in non-invasive PCa and LNCaP cells (Inoue et al., 2005; Giannoni et al., 2010). In our study, we used LNCaP and LNCaP-C81 cells to study the role of dioscin in PCa; we artificially reduced the uncontrollable endogenous or exocrine IL-6 expression levels from tumor cells that interfered with the results of this study. In addition, we found that dioscin inhibited cell proliferation and migration after applying IL-6. One study demonstrated that the IL-6/JAK/STAT3 pathway plays an important role in the tumor microenvironment and drug research. SHP1 has been identified as one of the most important negative regulators of STAT3 and increased expression of SHP1 inhibits STAT3 and present antitumor effects (Johnson et al., 2018). Another study demonstrated that SHP1 inhibits the proliferation and apoptosis of LNCaP cells as well as IL-6-treated offspring LNCaP-IL6^+^ cell lines, suggesting that SHP1 may be a therapeutic target for PCa (Tassidis et al., 2010b). In our study, dioscin promoted SHP1 phosphorylation and thus exhibited anticancer effects through the MAPK signaling pathway.

DHT also plays an important role in the development and progression of PCa. DHT activated ErbB-2 and Erk1/2-mediated PCa cell proliferation. In contrast, ErbB-2-specific inhibitors inhibit DHT-stimulated cell proliferation by blocking Erk1/2 (Muniyan et al., 2015). In the present study, dioscin inhibited the proliferation and migration of cells after DHT treatment and increased apoptosis. This role of dioscin in cells treated with DHT may also be related to the Erk1/2 and P38 MAPK signaling pathways.

Dioscin is a natural steroid saponin. Increasing evidence shows that dioscin exhibits anticancer properties against a variety of cancers (Wang et al., 2019). Zhou et al. (2019) screened 88 natural products and found that only dioscin significantly inhibited Skp2 and thus inhibited the progression of rectal cancer cells. After applying dioscin, A549, NCI-H446, and NCI-H460 lung cancer cells revealed DNA damage, mitochondrial structural changes, and blockage of the cell cycle in the S phase, indicating that dioscin increases lung cancer cell apoptosis (Wei et al., 2013). PCa changes from hormone dependence to hormone independence after treatment, which presents a significant challenge. It is thus of great importance to explore new drugs for treating hormone-independent PCa (Santer et al., 2015). Studies suggest that dioscin may be a potential treatment for PCa. Dioscin significantly inhibits cell viability, colony formation and motility and induces apoptosis in PC3 cells. Furthermore, dioscin reduces the levels of aldehyde dehydrogenase (ALDH) and CD133 (+)/CD44 (+) cells, suggesting that it also effectively inhibits PCa stem cells (Tao et al., 2017). In our study, dioscin effectively inhibited cell proliferation, reduced migration and exhibited antitumor effects on both hormone-sensitive PCa cells (LNCaP) and hormone-resistant PCa cells (LNCaP-C81).

The mechanism of how dioscin suppresses cancer remains unclear. Researchers have used inverse docking technology to screen potential cancer treatment drugs and discovered that only dioscin had the following properties for cancer treatment: it may be a TOP1 inhibitor, which halts the cancer cell cycle and DNA replication; it down-regulates epidermal growth factor receptor (EGFR) and EGF to inhibit growth of a vascular supply; it also has antiinflammatory activity by regulating the JNK signaling pathway to suppress cancer (Yin et al., 2015). Dioscin inhibits angiogenesis induced by the vascular endothelial growth factor receptor 2 (VEGF2) signaling and AKT/MAPK pathways, resulting in inhibition of cell proliferation, migration and invasion (Tong et al., 2014). The traditional herbal component verbascoside enhances phosphorylation of SHP1 by attenuating the TAK-1/JNK/AP-1 pathway, revealing an antiinflammatory effect by reducing the expression and activity of cyclooxygenase and nitric oxide synthase. However, depleting SHP1 eliminates the antiinflammatory effects of verbascoside (Pesce et al., 2015). Dioscin upregulates VEGF-A in osteoblast-like cells and increases angiogenesis to promote bone formation and fracture healing through hypoxia-inducible factor-1α activation of Src kinase, P38 MAPK and the Akt signaling pathways (Yen et al., 2005). Applying dioscin to A549 and H1299 lung cancer cells causes a dose-dependent increase in Erk1/2 and JNK1/2 activities, while reducing PI3K expression and Akt and mTOR phosphorylation (Hsieh et al., 2013). Our study demonstrated that dioscin promotes SHP1 phosphorylation, induces caspase-3 and Bad-related apoptosis and inhibits cell proliferation and invasion through the MAPK signaling pathway in PCa. In addition, we measured Ki67 expression in tumor tissues to assess tumor cell proliferation (Gerdes et al., 1984), but the Ki67 results were not different between the groups, suggesting that dioscin may not directly inhibit tumor cell proliferation. However, full-length caspase 3 (35 kD) is significantly inhibited by dioscin, whereas its cleaved forms (19 and 17 kD) increase significantly, indicating that dioscin may induce caspase 3-related apoptosis (Porter and Janicke, 1999; Min et al., 2004). We also found that dioscin promoted the expression of Bad, a pro-apoptotic BCL-2 family member, suggesting that dioscin induces cell apoptosis through the Bad pathway (Gajewski and Thompson, 1996). It should be noted that the antitumor effect of dioscin may be due to its hydrolysate diosgenin (Liu et al., 1995). Previous studies have revealed that in addition to diosgenin reducing cell proliferation and inducing apoptosis (Srinivasan et al., 2009), dioscin also inhibits angiogenesis, which inhibits tumor metastasis (Chen et al., 2015). Diosgenin reverses the multidrug resistance of cancer cells and make cancer cells sensitive to standard chemotherapy. Synthesis of diosgenin analogs and nano-formulations improve the anticancer efficacy and pharmacokinetic characteristics of the drug by enhancing the anticancer effects (Sethi et al., 2018). Therefore, future research on the active dioscin metabolites may be an important direction to study its anticancer effects.

SHP1 is a protein tyrosine phosphatase containing the Src homology 2 domain (SH2 domain). It has an oxidation sensitivity site and can be used as a target for antitumor therapy (Weibrecht et al., 2007). One study reported that the SHP1 protein is expressed in several non-lymphocytic cell lines, such as PCa, ovarian cancer and breast cancer cell lines, and that abnormal regulation of this protein causes abnormal cell proliferation and induces various cancers (Wu et al., 2003). Another study using PC3 cells revealed that SHP1 controls various factors necessary for normal functioning of the cell cycle through the PI3K-AKT pathway. Additionally, depleting SHP1 induced by siRNA causes G1 phase arrest, and SHP1 knockdown promotes nuclear localisation and p27 gene transcription (Rodriguez-Ubreva et al., 2010). SHP1 exhibits different expression levels and effects in different PCa cell lines. SHP1 is expressed at higher levels in LNCaP PCa cells than in PC3 cells and siRNA silencing of SHP1 increases LNCaP cell proliferation, while overexpression of SHP1 in PC3 cells decreases proliferation (Tassidis et al., 2010a). Clinical studies have shown that the expression of SHP1 in tumor cells is closely related to the timing of biochemical recurrence and clinical progression in PCa patients (Tassidis et al., 2010a). Reversing SHP1 gene silencing may be an attractive new strategy for cancer treatment (Zhang et al., 2005). Some natural antioxidants improve SHP1 expression. Several studies have reported that sylph verbascoside increases SHP1 expression, inhibits glioblastoma cell proliferation, migration and invasion and induces apoptosis. It also reduces tumor volume and growth in glioblastoma-planted nude mice (Jia et al., 2018). The SHP1 agonists SC-43 and SC-78 stimulate SHP1 to deactivate IL-6-induced STAT3 phosphorylation, thereby inhibiting colorectal cancer stem cells (Chung et al., 2018). Our study demonstrated that the antioxidant dioscin may have potential as a drug targeting SHP1. Dioscin promoted SHP1 phosphorylation and showed potential effects in the treatment of PCa.

In conclusion, the results of *in vitro* and *in vivo* studies have revealed that dioscin reversed IL-6 and DHT-stimulated PCa cell proliferation and invasion and increased apoptosis in these cells. These anticancer effects caused by dioscin may be due to its effects of increasing SHP1 phosphorylation and inhibiting the subsequent MAPK signaling pathway. Our results suggest that dioscin may be a potential drug to treat both androgen-sensitive and androgen-independent PCa.

## Data Availability Statement

The datasets generated for this study are available on request to the corresponding authors.

## Ethics Statement

The animal study was reviewed and approved by Ethics Committee of Fujian Provincial Hospital.

## Author Contributions

SHe wrote the manuscript. YG, XZ, and YW performed and designed the experiments. SHo and JY analyzed the data. HH performed the cell assays. QZ performed the WB assay. LY and TL performed *in vivo* experiments. YW, YG, and JY supervised the project.

## Funding

The study was supported by the Joint Funds for the innovation of science and Technology, Fujian province (2017Y9064); high-level hospital foster grants from Fujian Provincial Hospital, Fujian province, China (2019HSJJ29) the Project of Science and Technology Plan of Xiangtan, China (SF-CP20173004) and the Natural Science Foundation of Jiangsu Province (no.BK20160481).

## Conflict of Interest

The authors declare that the research was conducted in the absence of any commercial or financial relationships that could be construed as a potential conflict of interest.
