# Supplementary material for: Dioscin Promotes Prostate Cancer Cell Apoptosis and Inhibits Cell Invasion by Increasing SHP1 Phosphorylation and Suppressing the Subsequent MAPK Signaling Pathway
Source: Front Pharmacol. 2020 Jul 24;11:1099. doi: 10.3389/fphar.2020.01099 (PMC7394018; doi:10.3389/fphar.2020.01099)
Supplement: Supplementary file 1 [file DataSheet_1.docx]

**Supplementary figure legends**


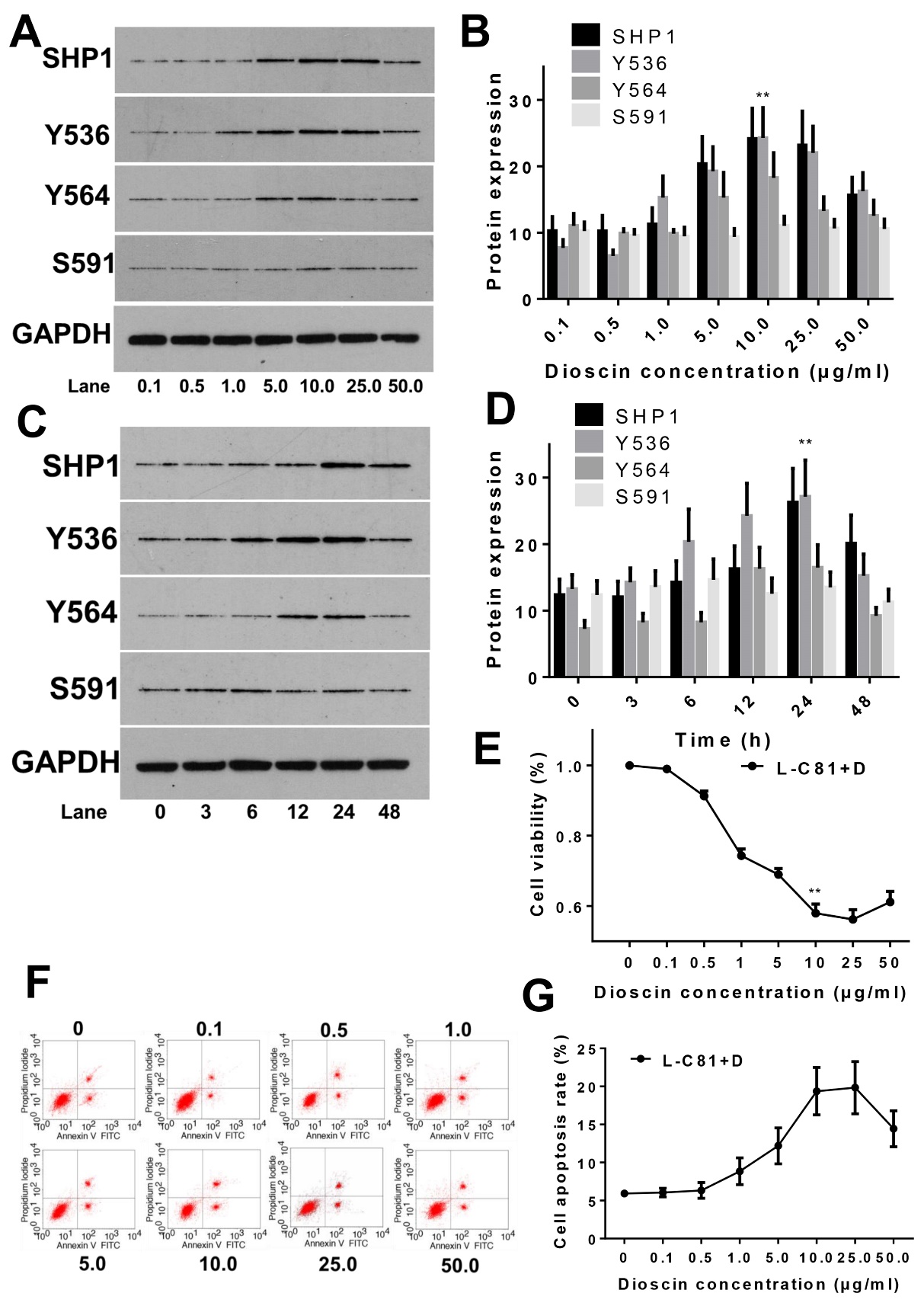


**Fig. S1** Screening for the optimal dioscin dose and duration for LNCaP-C81 cells.

(Fig. S1A and S1B) The expression levels of both SHP1 and p-SHP1 (Y536) are highest when LNCaP-C81 cells are treated with 10 μg/ml dioscin for 24 h (p <0.001). (Fig. S1C and S1D) The expression levels of both the SHP1 protein and p-SHP1 (Y536) are highest after 24 h of dioscin treatment (both p <0.001). Treatment with 10 μg/ml 24 h of dioscin can significantly inhibit LNCaP-C81 cell proliferation (Fig. S1E) and increase cell apoptosis (Fig. S1F and S1G) (both p <0.05).


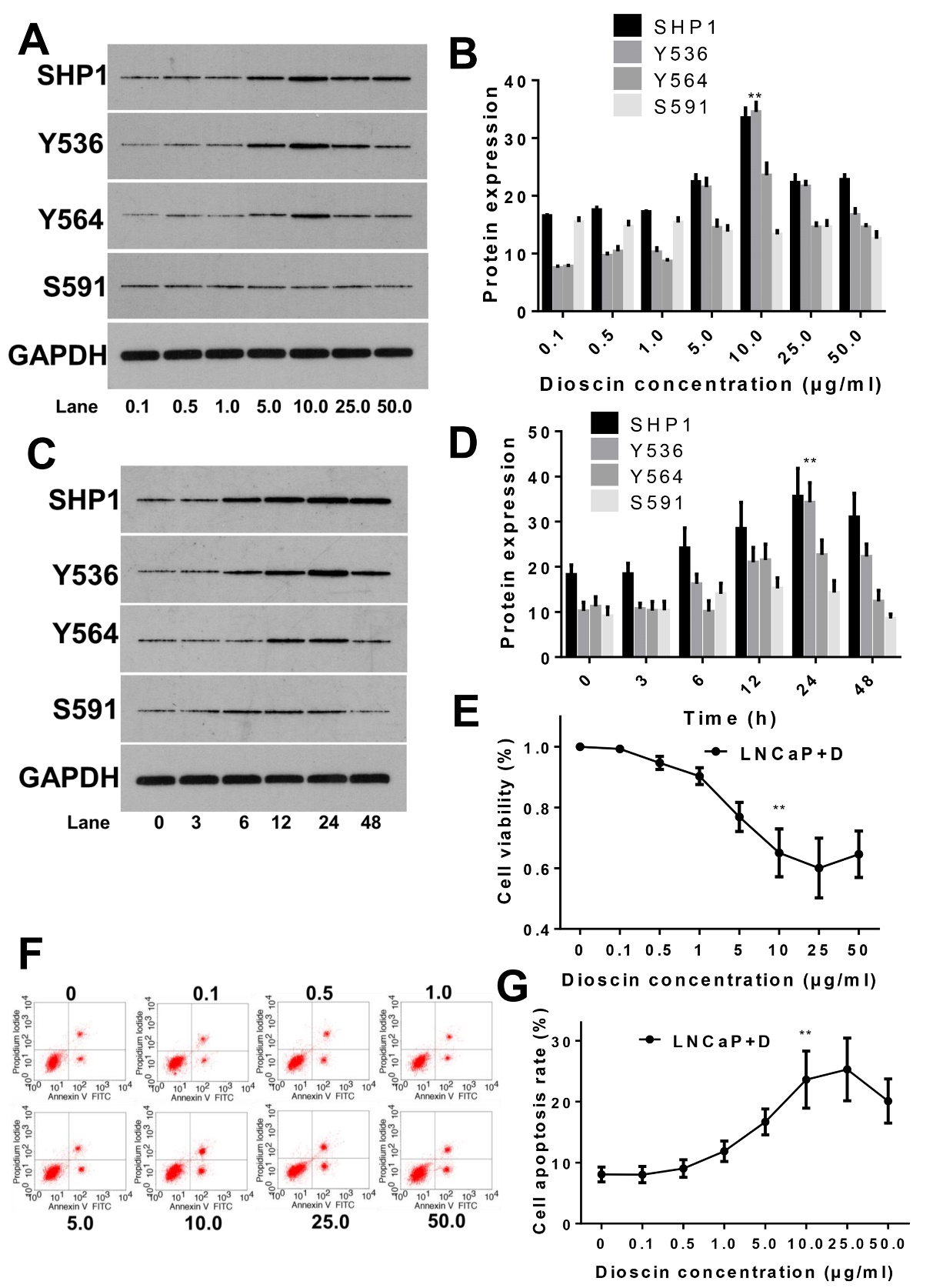


**Fig. S2** Screening for the optimal dioscin dose and duration for LNCaP cells.

(Fig. S2A and S2B) The expression levels of both SHP1 and p-SHP1 (Y536) are highest when LNCaP cells were treated with 10 μg/ml dioscin for 24 h (p <0.001). (Fig. S2C and S2D) The expression levels of both SHP1 protein and p-SHP1(Y536) are highest after 24 h of dioscin treatment (both p <0.001). Application of 10 μg/ml of dioscin for 24 h can significantly inhibit LNCaP cell proliferation (Fig. S2E) and increase cell apoptosis (Fig. S2F and S2G) (both p <0.05).


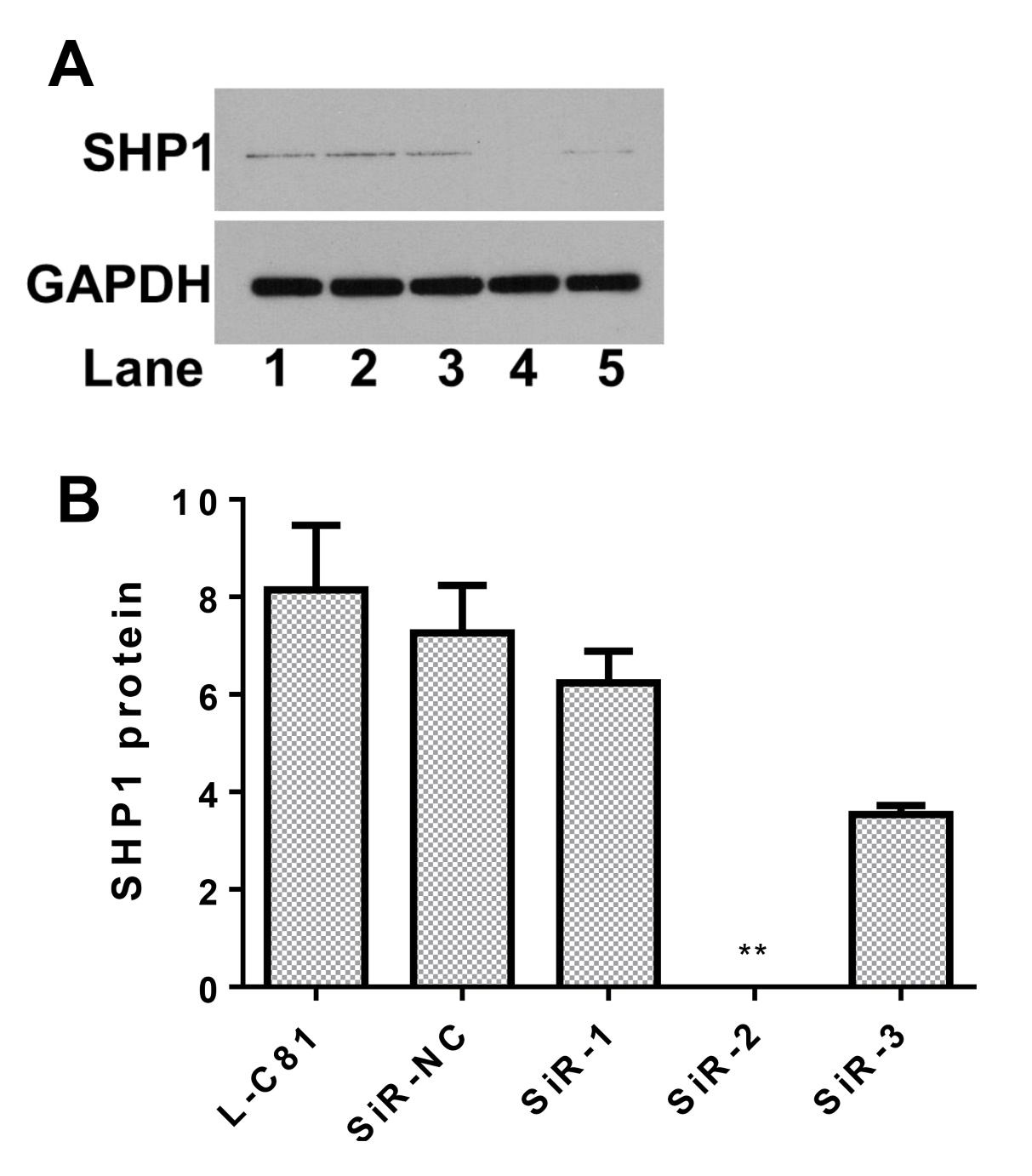


**Fig. S3** Dioscin regulates SHP1 to reverse IL-6-induced LNCaP-c81 cell proliferation and invasion

(Fig. S3A and S3B) SiR-2 can completely block the expression of SHP1.


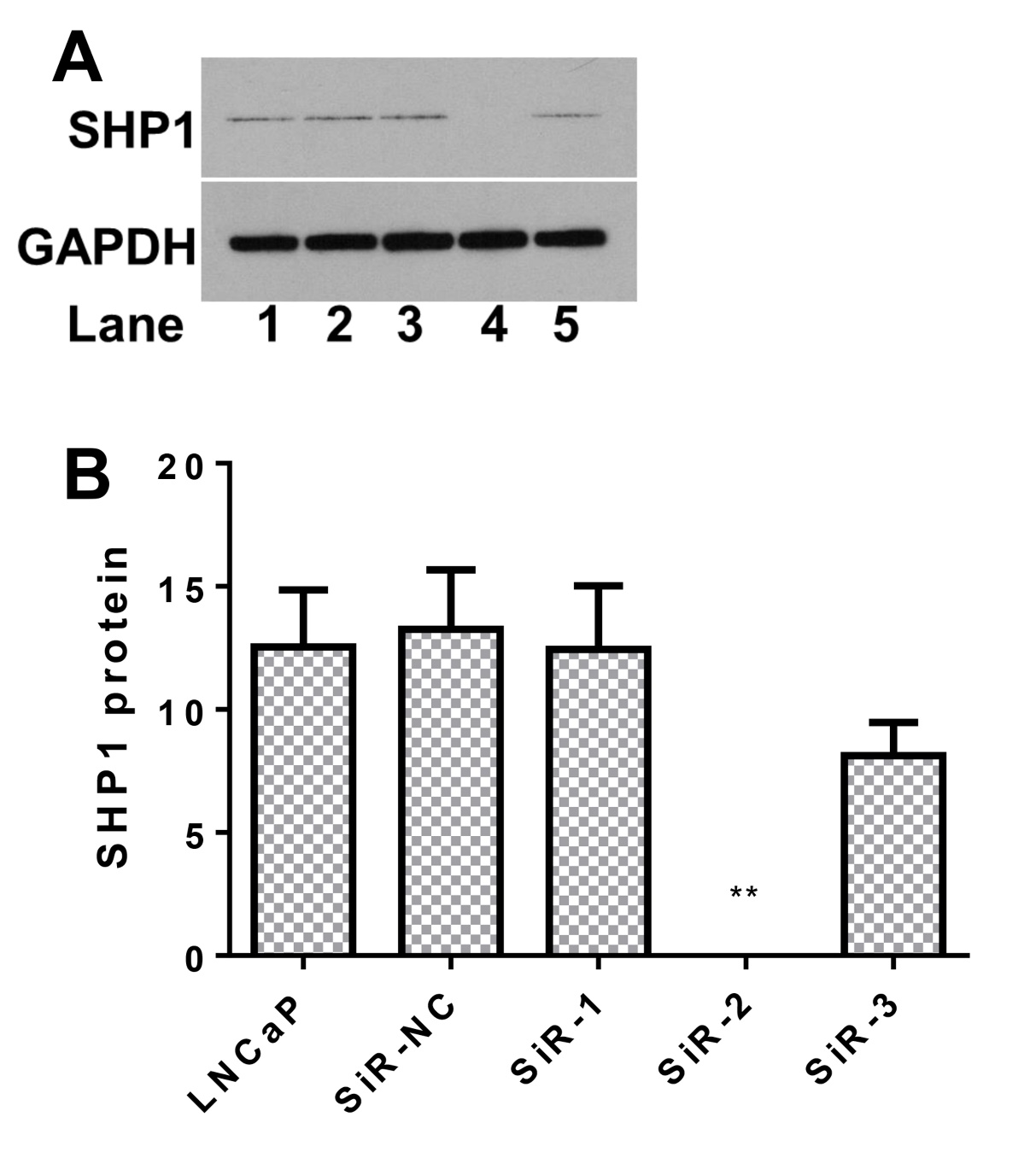


**Fig. S4** Dioscin regulates SHP1 to reverse IL-6-induced LNCaP cell proliferation and invasion

(Fig. S4A and S4B) SiR-2 can completely block the expression of SHP1.
