# Supplementary material for: Dioscin Promotes Prostate Cancer Cell Apoptosis and Inhibits Cell Invasion by Increasing SHP1 Phosphorylation and Suppressing the Subsequent MAPK Signaling Pathway
Source: Front Pharmacol. 2020 Jul 24;11:1099. doi: 10.3389/fphar.2020.01099 (PMC7394018; doi:10.3389/fphar.2020.01099)
Supplement: Supplementary file 2 [file Table_1.docx]

S Table-1 Primary antibodies for western blot.

| **Primary antibodies** | **MW (kDa)** | **Dilution** | **Company / Catalog** |
| --- | --- | --- | --- |
| SHP1 | 68 | 1:1000 | abcam, ab32559 |
| p-SHP1(Y536) | 68 | 1:1000 | abcam, ab51171 |
| p-SHP1(Y564) | 68 | 1:1000 | abcam, ab79294 |
| p-SHP1(S591) | 68 | 1:1000 | abcam, ab41436 |
| ERK1/2 (p-T202/T204) | 42/44 | 1:200 | sab, 12082 |
| p-ERK 1/2 Antibody (T177) | 42/44 | 1:500 | SANTA, sc-16981-R |
| P38 MAPK(p-T182) | 43 | 1:500 | sab, 11253 |
| P38 MAPK(p-T180) | 43 | 1:500 | sab, 11252 |
| Akt (p-T326) | 60 | 1:500 | sab,12126 |
| Akt (p-S129) | 65 | 1:500 | sab,11914 |
| Caspase 3 | 35/19/17 | 1:500 | Abcam, ab90437 |
| Bad | 23 | 1:2000 | Abcam, ab32445 |
| GAPDH | 37 | 1:500 | SANTA, SC-365062 |
